# Using Heating and Cooling Presses in Combination to Optimize the Consolidation Process of Polycarbonate-Based Unidirectional Thermoplastic Composite Tapes

**DOI:** 10.3390/polym15234500

**Published:** 2023-11-23

**Authors:** Janos Birtha, Eva Kobler, Christian Marschik, Klaus Straka, Georg Steinbichler

**Affiliations:** 1 Institute of Polymer Injection Moulding and Process Automation, Johannes Kepler University Linz, Altenbergerstraße 69, 4040 Linz, Austria; 2 Competence Center CHASE GmbH, Altenbergerstraße 69, 4040 Linz, Austria

**Keywords:** thermoplastic composites, consolidation, optimization, unidirectional tapes

## Abstract

The main aim of this work was to optimize the consolidation of unidirectional fiber-reinforced thermoplastic composite tapes made of polycarbonate and carbon fibers using a heating press and a cooling press in combination. Two comprehensive studies were carried out to investigate the impact of process settings and conditions on the quality of the consolidated parts. The initial screening study provided valuable insights that informed the design of the second study, in which the experimental design was expanded and various modifications, including the implementation of a frame tool, were introduced. The second study demonstrated that the modifications in combination with a high heating press temperature and elevated heating and cooling pressures successfully achieved the desired goals: the desired thickness (2 mm), improved bonding strength (23% increase), and reduced void content (down to 4.64%) in the consolidated parts.

## 1. Introduction

Thermoplastic composites are prime candidates for the design of lightweight components. Compared to their thermoset counterparts, their advantages include that they can be remelted multiple times, are recyclable, and they offer high damage resistance and excellent vibration dampening [1,2]. One way of manufacturing or locally reinforcing structural plastic components is to use unidirectional (UD) fiber-reinforced thermoplastic tapes. They provide part designers with a high degree of flexibility, since they can be freely oriented and layered in an automated manner. Alongside the automated tape placement (ATP) process, compression molding using a hot plate to consolidate UD tapes is gaining in popularity due to the shorter cycle times required.

The consolidation process involves melting of the thermoplastic matrix of the UD tapes under compression, which causes the viscosity of the polymer to decrease. This allows the voids due to asperities of the tapes to be filled in. Once in full contact, the molecular chains of the matrix material can diffuse across the interface, which—after cooling—achieves bonding between the tapes [3]. The quality of the consolidated tape stack strongly depends on the processing conditions, such as temperature, pressure, and time. Producing a void-free, well-bonded part therefore requires finding optimal process settings.

The rich literature describes a variety of experimental approaches to optimizing the consolidation of polyether ether ketone (PEEK)-based thermoplastic composites using different processes. Khan et al. [4,5] used the ATP process to monitor the temperature profile below the first and seventh layer using thermocouples during consolidation, and to optimize various process settings based on the void content, the density, and the interlaminar shear strength using lap-shear tests. Similarly, Sonmez et al. [6] used PEEK with the ATP process to find optimal process settings by observing the peak residual stress, thermal degradation, and the degree of bonding. With three different processes, namely the vacuum-bag-only in oven (VBO) approach, laser-assisted automated fiber placement (AFP), and using a hot press, Saenz-Castillo et al. [7] measured the void content, the in-plane shear strength, and interlaminar shear stress (ILSS) to assess the optimal settings for producing PEEK-based composites.

The majority of studies have focused on PEEK matrices, while other materials have received less attention. Saffar et al. [8] used the VBO technique to investigate the bonding quality of polyetherketoneketone (PEKK)-based composites. Hoang et al. [9] performed a large-scale study employing the same type of material, but post-consolidated their parts using four different methods, including in situ consolidation, annealing, VBO, and using a hot press. Optimization of the consolidation process based on void content and mechanical properties in an autoclave with carbon/polyphenylene sulfide (CF/PPS) was investigated by Patou et al. [10]. Further materials, such as polyaryletherketone (PAEK) [11], polyamide-6 [12], and polypropylene-based [13] thermoplastic composites have also been used to optimize various processing techniques. To the best of our knowledge, research based on the consolidation of polycarbonate-based (PC) composites is scarce. Notably, Borowski et al. [14] used carbon-fiber-reinforced polycarbonate (PC/CF) to optimize their additive manufacturing-based in situ consolidation process by measuring the flexural strength and the porosity of the finished part. Asséko et al. focused on the temperature development during the joining of glass-fiber-reinforced polycarbonate (PC/GF) and confirmed that—even above the glass-transition temperature—perfect cohesion was not achieved [15].

Compared to the ATP and automated fiber-placement (AFP) processes, the consolidation of thermoplastic composites using a hot press has gained less attention. Schnell et al. [13] determined the robustness of the hot press process by the bonding quality of polypropylene (PP)/glass prepregs. They also used thermocouples to measure the temperature of the hot plates and at the interface between the prepregs to assess the stability of the process. In a study by Almeida et al. [16], PEEK laminates were used to assess the degradation of the matrix and the type of voids appearing in the semi-finished product. As previously mentioned, Saenz-Castillo et al. [7] used various processing techniques, including a hot press. In their study, they varied the temperature and the pressure of a hot press to assess how the void content and the ILSS are influenced by changing the process settings.

Most of these studies used the void content or interlaminar strength to assess the quality of the final product. Other mechanical tests used for optimization include the double cantilever beam test [17], the four-point bending test [18], and the mandrel peel test [19]. In the case of semi-crystalline matrix materials, the degree of crystallinity is also an important quality parameter [20,21,22]. By means of process modeling and experimental investigations, Sonmez et al. [3,6,23] measured the residual stress, among other quality parameters, to optimize the ATP process. The investigation of fiber waviness development during the consolidation process has recently started to attract attention, as it leads to a decline in compressive strength and surface quality [24,25,26]. Finally, the change in laminate thickness and the resulting squeeze flow has also been considered in some studies [27,28].

The main objective of this work was to optimize the consolidation process of PC/CF UD tapes using a combination of a hot and a cold press in three consecutive studies. The choice of material was based on the selection of an amorphous matrix-based thermoplastic composite, suitable for potential use in interior automotive applications. The overall goal was to obtain well-bonded, void-free consolidated parts made from 12 individual layers with a total thickness of 2 mm. From an optimization perspective, this involved (i) maximizing bond strength, (ii) minimizing void content, and (iii) achieving a 2 mm thickness to match the wall thickness of the injection molding machine’s tool in the downstream process for an arbitrary amorphous matrix-based thermoplastic composite. First, consolidation experiments were performed to assess the thermal behavior of the core layer of the stack after changing the set temperature in both the heating and the cooling presses. For this purpose, Type K thermocouples were positioned in the center of the stack, and the information gathered was used to determine the cycle time. Second, a screening test was carried out to evaluate industrially relevant process windows. To this end, we investigated the influence of various process parameters—the cycle time, temperature, and pressure of the heating and cooling presses—on the quality of the consolidated part in terms of bonding strength, thickness, density, and warpage. In addition, we examined the change in part area after consolidation (as a measure of squeeze flow). Third, informed by the results of the screening test, we implemented a frame tool. Applying the findings of the previous analyses, we used a central composite design to identify optimal process parameters that produce the highest quality in terms of bonding, void content, thickness, and warpage.

## 2. Experimental

### 2.1. Material

We used Maezio CF GP 1003T UD tapes made from PC/CF with a fiber volume content of roughly 44% (according to the material supplier’s data sheet) in our consolidation experiments provided by Covestro AG (Leverkusen, Germany). Table 1 shows an overview of the properties of the matrix and fiber materials. The tape stacks were positioned and spot-welded in a tape-laying cell based on the pick-and-place principle [29]. In each case, 12 layers of UD tapes were arranged in a stacking sequence of [0°/90°/0°/90°/0°/90°]s. The nominal dimensions of the tape stack were 230 × 150 × 2.1 mm. However, a previous study focusing on measuring the thickness of UD tapes at 648 different positions revealed an average tape thickness of 0.186 ± 0.0073 mm. This analysis confirms that stacking 12 layers of these UD tapes should yield a total thickness greater than 2.1 mm.

### 2.2. Experimental Set-Up

**Figure 1:**
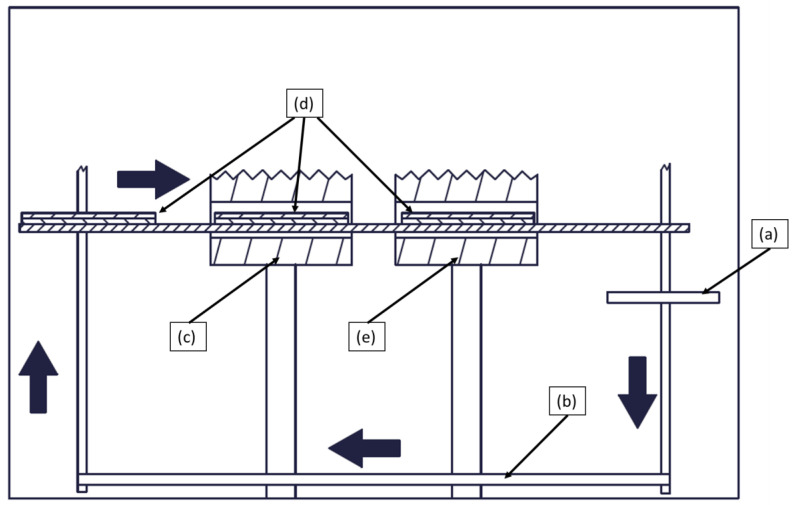
Consolidation unit used in the experiments: (**a**) transport system, (**b**) shuttle system, (**c**) heating press, (**d**) steel plate tools, (**e**) cooling press.

The tape stacks were consolidated in a FILL SM-03 consolidation unit, as shown in Figure 1. The unit consists of two hydraulic presses, namely a heating press and a cooling press, along with a transport system for the material being processed. The tape stack is initially placed between two 5 mm steel tool plates (Figure 1a), which are first moved (Figure 1b) to the heating press (Figure 1c). In this stage, the lay-up is subjected to elevated temperatures above the glass-transition (T_g_) (in case of amorphous polymers) or melting (T_m_) temperature (in case of semi-crystalline polymers) of the matrix, while pressure is applied for a predetermined period. The steel plates (Figure 1d) carrying the molten material are then transported to the cooling press (Figure 1e) within approximately 5 s, where it is cooled to below the T_g_ or T_m_ of the matrix. Once the fully automatic process is completed, the consolidated plate can be retrieved by lifting the top steel plate using sucker pins and a mechanical locking mechanism.

The heating press can reach a maximum temperature of 450 °C and apply a maximum force of 25 kN. In contrast, the cooling press has a maximum operating temperature of 140 °C and can exert a maximum force of 290 kN. These specifications define the upper temperatures and forces that the presses can achieve and maintain during operation. These limits also define the operation of the consolidation unit: in the heating press, minimal pressure is applied to prevent excessive squeeze flow, while in the cooling press a much higher force is applied to finalize the consolidation step.

Furthermore, pressure sensors installed at the hydraulic accumulators indirectly measure the force exerted on the parts. They are used to determine the time required to reach the maximum pressure during the consolidation process.

### 2.3. Experimental Design

In the first set of experiments, the preliminary tests, the temperature in the core of the laminate at 200 °C, 250 °C, and 300 °C heating press temperature and 60 °C cooling press temperature was assessed using thermocouples. These were positioned between the 6th and 7th layers of the tape stack to measure the minimum amount of time it takes for the core to reach the set temperature. In both presses, the pressure was kept low (1 bar and 10 bar for the heating and cooling presses, respectively) to ensure that the sensors remain intact.

Figure S1 illustrates the temperature evolution within the core of the samples at various heating press temperatures for the settings investigated. Heat is conducted from the plates via the outer layers to the core layer, which takes time. Consequently, we sought to investigate the time-dependent temperature behavior of the core layer. The heating process is completed when the core layer has reached its required temperature. As anticipated, higher set temperatures of the plate surfaces require more time for the core temperature to reach the set temperature, with approximately 104 s for 200 °C, 138 s for 250 °C, and 168 s for 300 °C. These values constitute the lower limits of the experimental design, representing the minimum periods of time required to reach the desired core temperature.

Furthermore, an additional test was conducted as part of the optimization trial, where two specimens were produced using the lowest (250 °C heating press temperature, 60 °C cooling temperature, 1 bar heating pressure, 10 bar cooling pressure) and highest (325 °C heating press temperature, 100 °C cooling press temperature, 6.5 bar heating pressure, 85 bar cooling pressure) process settings possible. Similarly, a thermocouple was positioned in the core layer of both stacks to monitor the temperature changes during consolidation. Pressure sensor data were additionally collected by an HBM data-recording device (QuantumX CS22B-W, HBM, Darmstadt, Germany) and analyzed in these experiments.

In the second set of experiments, the screening test, a definitive screening test design, was employed with 13 different settings. An overview of the experimental design is given in Table S1. The parameters were selected with the aim to (i) avoid thermal degradation of the matrix while (ii) covering a broad range of physical conditions. The consolidation pressures exerted on the parts were based on the set force and their nominal area. The holding time corresponds to an additional period that extends beyond the time necessary for the core of the part to reach the set temperature. This holding time parameter was established based on findings from preliminary tests (Figure S1). Twelve plates were produced within one consolidation parameter setting. No flow restrictions were applied to the tape stack under consolidation.

To analyze the quality of the consolidated parts, destructive and non-destructive methods were employed. Due to spatial constraints of some measurement systems, we used smaller plates (20 × 10 mm^2^, obtained by water cutting) to analyze thickness, density, and apparent shear strength (ASS). The remaining parameters (e.g., warpage) were measured using the whole plate.

**Figure 2:**
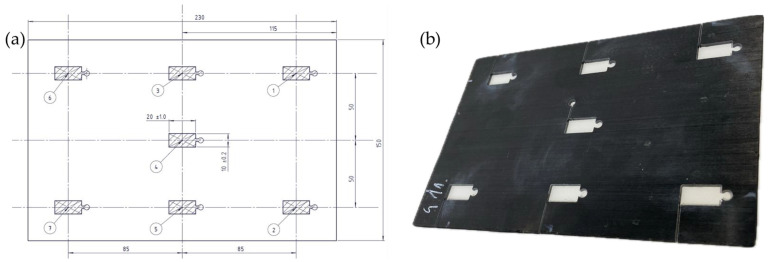
(**a**) Location and numbering of the small samples used in the screening and optimization trials; (**b**) photograph illustrating a consolidated plate with small samples cut out from it.

Position, size, and numbering of the cut samples are shown in Figure 2. The metrics used to analyze the quality of consolidation were: (i) projected area, (ii) thickness, (iii) warpage, (iv) density, and (v) ASS.

In the third set of experiments, the optimization test, several adjustments to the experimental design and setup were made based on the findings of the previous studies, such as expansion of the design space and introduction of a frame tool. A split-plot central composite design consisting of 26 different settings with four factors and multiple levels was employed, with the setting at the center point performed three times. Three plates were produced for each setting. In this new design, the holding time as an influencing factor was replaced by a constant cycle time of 3 min for both presses. Split-plot designs allow factors to be set that cannot be changed within an experimentally reasonable time frame, which imposes limitations on the randomization of the experiment’s execution order. In particular, the temperature of the heating press—changing of which requires a significant amount of time—was considered as such a factor. The experimental design of the optimization test is shown in Table S2. Selection of the settings was guided by the insights gained from the screening test (see Section 3.1) and aimed to establish a configuration in which both the temperatures and pressures were maximized in relation to the recommended maximum processing temperatures and forces of the consolidation unit.

**Figure 3:**
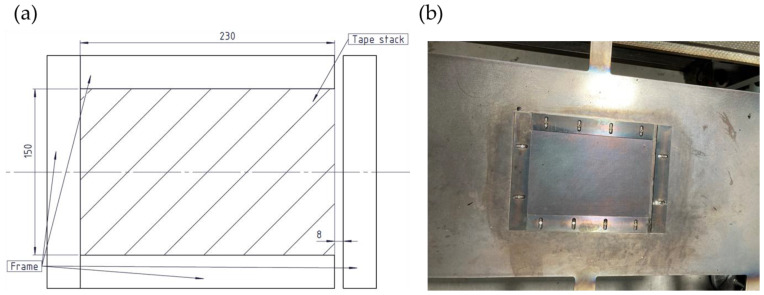
(**a**) Frame tool used to manufacture the samples for the optimization trials. The hatched area indicates the position of the tape stack under consolidation; (**b**) visual representation of the frame tool.

A frame tool with a thickness of 1.9 mm was installed between the two steel plates, as can be seen in Figure 3. A small opening was intentionally incorporated on the right side of the frame tool. Screening trials revealed that, when subjected to high pressure, the matrix material exhibited excessive flow in multiple directions, which made part removal difficult. Forcing the flow of the matrix material in one predefined direction made part handling easier. To assess the quality of the manufactured plates, (i) thickness, (ii) warpage, (iii) void content, and (iv) ASS were investigated. In analogy to the screening test, thickness, void content, and ASS measurements were performed using smaller samples. However, only areas 1, 4, and 7 (see Figure 2) were taken under consideration due to the high number of plates produced during the optimization trials. A summary of both experiments can be seen in Table 2.

To analyze the influence of process settings on quality parameters, main influence graphs, cube plots, and surface plots were produced. To confirm the statistical significance of the results, we applied the analysis of variance (ANOVA) statistical method (with 95% statistical confidence) using the Design Expert software package (version: 22.0.4 64-bit). The resulting ANOVA table provides a summary regarding the statistical significance of factors through *p*-values. These *p*-values are calculated based on the sum of squares, which represents the squared differences between the overall average and the observed variation, degrees of freedom (df), denoting the number of estimated parameters used for computing the sum of squares, and the F-value, which serves as a test for comparing the calculated mean square to the residual mean square. A factor is considered influential with 95% statistical confidence when the corresponding *p*-value is below 0.05. The design model used in the screening trial was a reduced quadratic model that considered the main effects and the interaction between heating press temperature and pressure. In contrast, the optimization trial employed a quadratic model that incorporated all two-factor interactions. The summary of the ANOVA tables that served as a basis for interpretation of influencing factors can be found in the Supplementary.

### 2.4. Measurement Methods

#### 2.4.1. Projected Area

**Figure 4:**
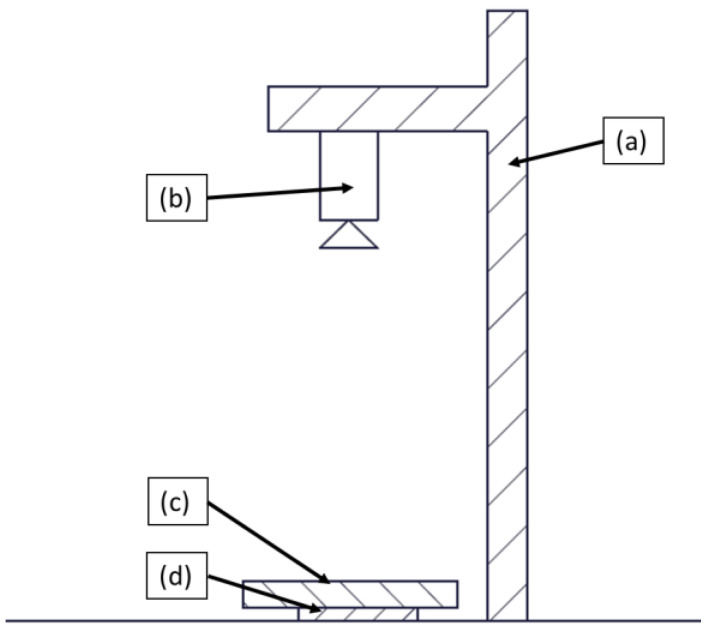
Schematic drawing of the experimental setup of the area measurement: (**a**) stand, (**b**) optical camera, (**c**) glass plate, and (**d**) specimen.

To measure the projected area of plates manufactured during the screening test, a high-resolution camera with 64 megapixels was used to record images. The camera was mounted on a stand, positioned above the consolidated plates placed on the ground. However, due to significant warpage observed in these analyses, the samples were flattened using a 500 × 500 × 6 mm^3^ anti-reflective white glass. This glass helped to minimize distortions and ensured accurate measurement of the projected area. The schematic drawing of the setup is given in Figure 4.

To determine the projected area of the parts, two prerequisites had to be fulfilled. First, the pictures captured had to exhibit sufficient contrast to allow thresholding of the part from the background. This was achieved by placing the black part against a white background. Second, the scale of pixels–mm had to be determined. To achieve this, three “calibration” pictures of the same 200 × 150 mm^2^ part were taken. By carefully selecting the longitudinal length and converting the pixel distance to millimeters, a scale was obtained. To allow fast thresholding and measurement of the projected areas, the ImageJ software (version: 1.53e) was employed, which provides automated thresholding and enables quick computation of the areas.

#### 2.4.2. Thickness

Thickness measurements of the consolidated plates were conducted using a micrometer with a precision of ±0.01 mm. Due to the limited reach of the measurement device, the small (i.e., cut) samples specified in Figure 2 were analyzed in both the screening and optimization trials.

#### 2.4.3. Warpage

**Figure 5:**
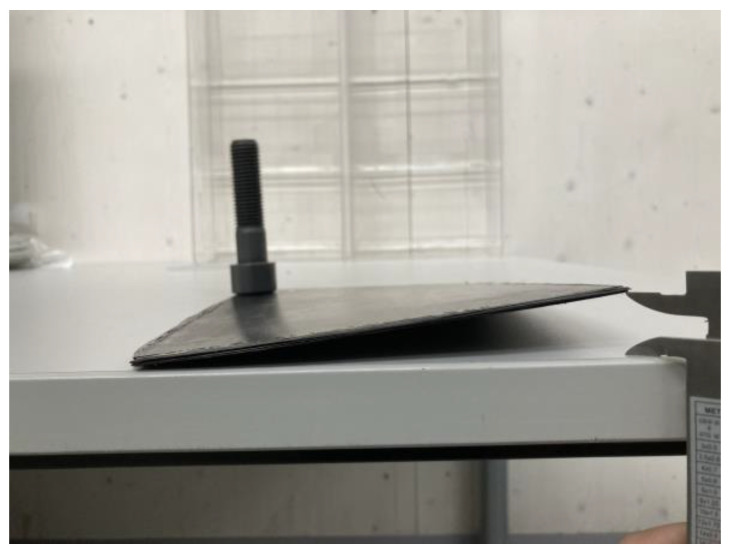
Warpage measurement of a consolidated plate.

To assess the level of residual stress accumulated in a plate during the consolidation process, warpage tests were carried out. The test involved placing a part on a flat surface, securing it at one corner, and measuring the degree of lifting of the part using a caliper, as illustrated in Figure 5.

#### 2.4.4. Density and Void Content

The density of the small plates extracted from the consolidated plates (see Figure 2) was determined according to ASTM D792-13 [30] (November 2013). In addition, for the optimization tests, the void content was determined in accordance with ASTM D2734-16 [31] (December 2016). To obtain the fiber content, the matrix was removed by burning in a Gero HTK 8 high-temperature furnace according to ASTM D2584 [32]. Based on results from preliminary tests, the furnace was heated at a rate of 10 °C per 10 min until a temperature of 900 °C was reached. The parts were then held in the furnace at an isothermal temperature of 900 °C for one hour and subsequently cooled. Nitrogen was used throughout the process at a flow rate of 250 L/h. To obtain the void content, the following equation was used:

(1) $$V_{v}=100-\left(\frac{M_{f}}{M_{i}}\times 100\times \frac{\rho_{c}}{\rho_{r}}\right)-\left(\frac{M_{i}-M_{f}}{M_{i}}\times 100\times \frac{\rho_{c}}{\rho_{m}}\right),$$

where $M_{f}$ is the weight of the fiber that is retained after the matrix burn-off test, $M_{i}$ is the initial weight of the composite, $\rho_{c}$ is the density of the composite, $\rho_{r}$ is the density of the reinforcement, and $\rho_{m}$ is the density of the matrix material [9]. The densities of matrix and fiber were 1.19 g/cm^3^ and 1.8 g/cm^3^, respectively.

#### 2.4.5. Apparent Shear Strength

The ASS tests were performed in accordance with the ISO 14130 [33] (1997) standard to evaluate the bonding strength of small samples. An MTS 852 Test Damper System with a 10 kN loading cell was used with a 1 mm/s displacement rate. The shear strength of the specimens was determined by considering the first maximum force achieved during the testing procedure.

## 3. Results and Discussion

### 3.1. Screening Test

#### 3.1.1. Projected Area and Thickness

**Figure 6:**
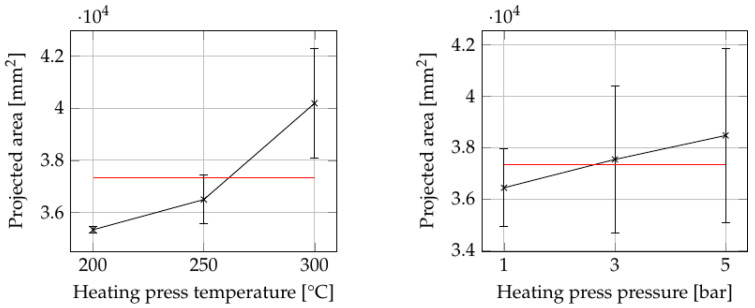
The consolidation settings with the greatest influence on the projected area of the plates as summarized in Table S1. The red line indicates the grand average of all values.

**Figure 7:**
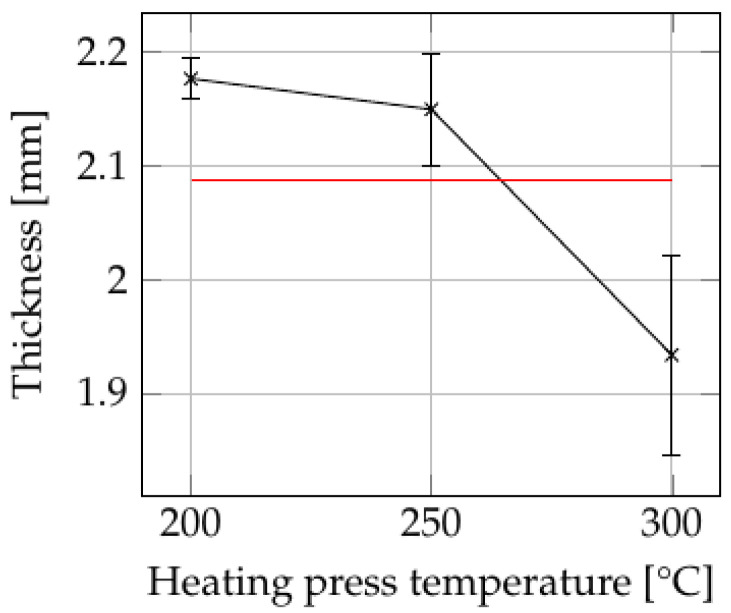
The influence of heating press temperature on the thickness of the plates. The red line indicates the grand average of all values.

Figure 6 illustrates the consolidation settings (see Table S1) with the greatest influence on the projected area of the plates, while Figure 7 shows the effects of consolidation settings that led to significant variations in the thickness of parts. ANOVA reveals that both temperature and pressure of the heating press had a statistically significant influence on the projected area, with calculated *p*-values below 0.05 (see Table S3). Regarding the thickness, the heating press temperature showed a significant effect with a *p*-value of 0.0014. However, no other process parameters exhibited a discernible influence on either area or thickness.

**Figure 8:**
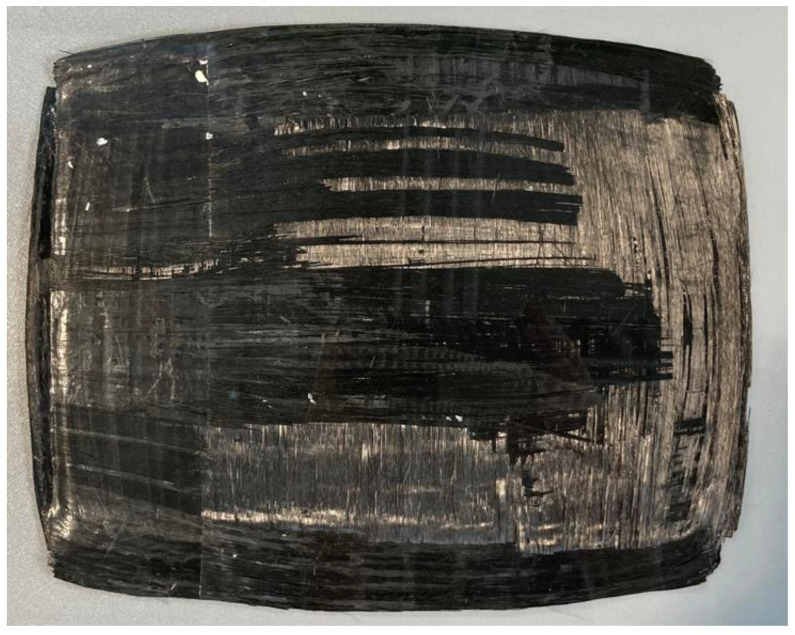
A split-open sample produced at 300 °C heating press temperature and 5 bar heating press pressure. The fibers at the sides deviate from their original orientation.

At a heating press temperature of 200 °C, the plate showed no observable deviation from its initial size based on the projected area. At higher heating press temperatures, particularly at 300 °C, the plate exhibited an increased projected area and reduced thickness. This behavior can be attributed to the material’s ability to flow freely in all directions, resembling a squeeze flow mechanism. Figure 8 shows a split open sample produced at a heating press temperature of 300 °C and a heating press pressure of 5 bar. It is evident that the squeeze flow not only significantly reduced the thickness, but also distorted the fiber structure of the plate. On the sides, the fibers appear slightly bent, reflecting the flow behavior of the matrix material. Consequently, a decrease in mechanical performance is expected in these areas.

**Figure 9:**
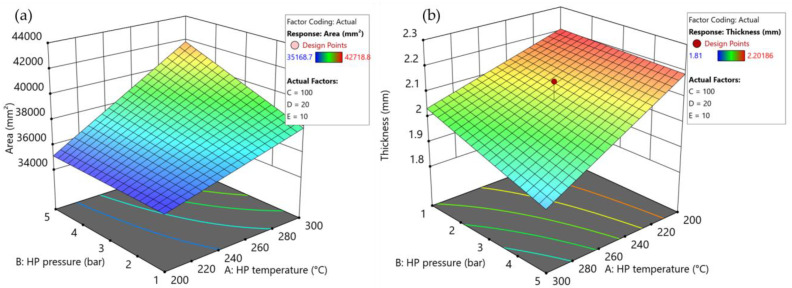
Surface plots showing the combined influence of heating press (HP) temperature and pressure on (**a**) the projected area and (**b**) the thickness in the screening test as summarized in Table S1. Set values for the remaining parameters: cooling press temperature: 100 °C (factor C); cooling press pressure: 20 bar (factor D); holding time: 10 s (factor E).

The combined influence of heating press temperature and pressure is illustrated in Figure 9 for (a) area and (b) thickness. The ANOVA analysis reveals statistically significant effects of these two factors on the projected area and thickness. The influence of heating press pressure increased with increasing temperature. However, for the thickness, the *p*-value of 0.19 indicates no statistical significance. This lack of significance may be attributable to the considerable variations in tape thickness, as previously indicated. Nevertheless, it is evident that at higher heating press temperatures, the pressure applied by the heating press has an impact on the thickness achieved. Other two-factor interactions either lack statistical significance or are confounded with other factors, thus limiting the scope of analysis in this study.

#### 3.1.2. Warpage

**Figure 10:**
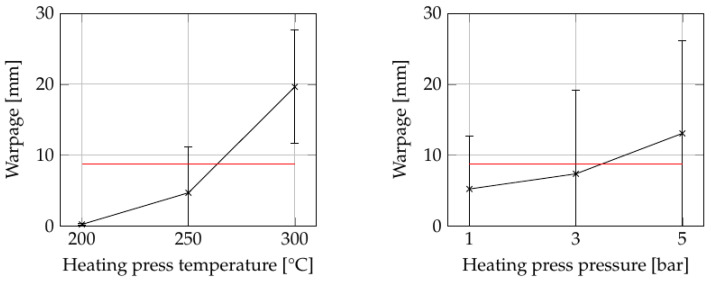
The influence of consolidation settings as summarized in Table S1 on the warpage of the plates. The red lines indicate the grand average of all values.

**Figure 11:**
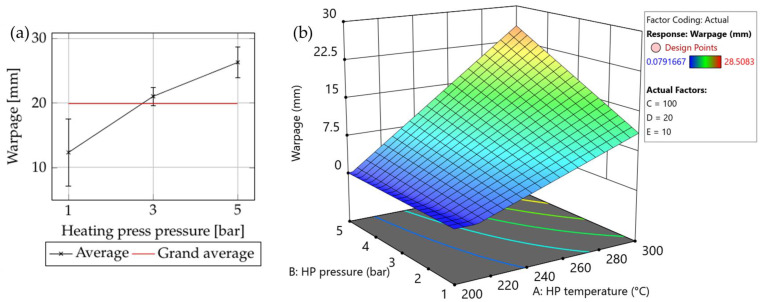
Detailed examination of the warpage results in the screening test: (**a**) the influence of heating press pressure at 300 °C heating press temperature; (**b**) surface plot showing the combined influences of heating press (HP) temperature and pressure on the warpage in the screening trial as summarized in Table S1. Set values for the other parameters: cooling press temperature: 100 °C (factor C); cooling press pressure: 20 bar (factor D); and holding time: 10 s (factor E).

Figure 10 illustrates the consolidation settings (see Table S1) with the greatest influence on the warpage of parts. Again, ANOVA showed that the influence of heating press temperature and pressure were significant, with *p*-values of 0.0005 and 0.033, respectively (see Table S5). Although the effect of the heating press pressure may not be immediately apparent in the main influence graphs, a closer examination of the results shown in Figure 11 reveals its significance at 300 °C.

As the part is squeezed during consolidation, the internal structure of the part also changes, as demonstrated in Figure 8. Increasing the heating press parameters leads to greater squeeze flow, resulting in a distorted laminate structure and subsequent warpage. Further, the dynamic nature of the process is noteworthy. During the screening experiments, the specimens were heated to the desired temperature within 2–3 min (depending on the set temperature) and subsequently cooled within the same time frame. We hypothesize that the rapid thermal cycling during the process may have induced higher levels of residual stress in the plate, thereby increasing warpage in the final part.

#### 3.1.3. Density

**Figure 12:**
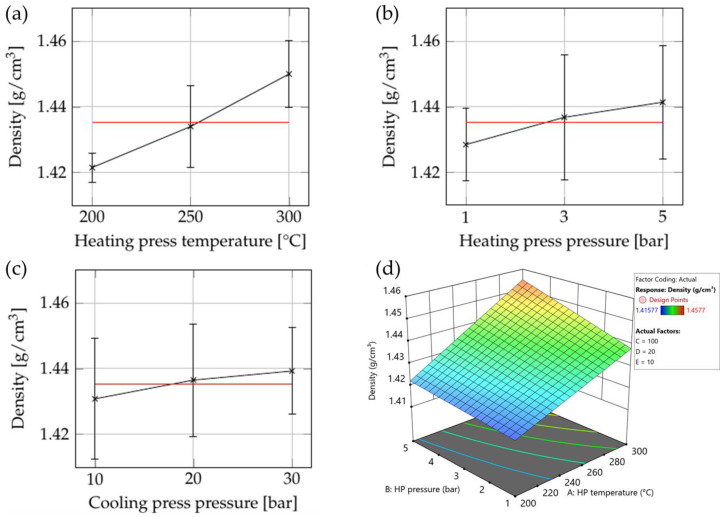
The influence of (**a**) heating press temperature, (**b**) heating pressure, and (**c**) cooling press temperature on the density of the plates (the red lines indicate the grand average of all samples). (**d**) Surface plot showing the combined influences of the heating press (HP) temperature and pressure on the density in the screening trial. Set values for the other parameters: cooling press temperature: 100 °C (factor C); cooling press pressure: 20 bar (factor D); and holding time: 10 s (factor E).

The influence of the consolidation settings (see Table S1) on the density of the plate is illustrated in Figure 12. ANOVA shows that the heating press temperature had the greatest impact, followed by the heating press pressure and the cooling press pressure (see Table S6). There is a slight correlation between the first two, but it is not statistically significant (see Figure 12d). With increasing heating press temperature and pressure, the matrix material exhibited enhanced flowability, which allowed the gaps and voids between the layers of the composite tape stacks to be filled more effectively. The high standard deviation of densities indicates that the press was unable to apply uniform pressure to the part. Additional investigation revealed that this issue was particularly prominent at cooling press pressures of 10 and 20 bar.

#### 3.1.4. ASS

**Figure 13:**
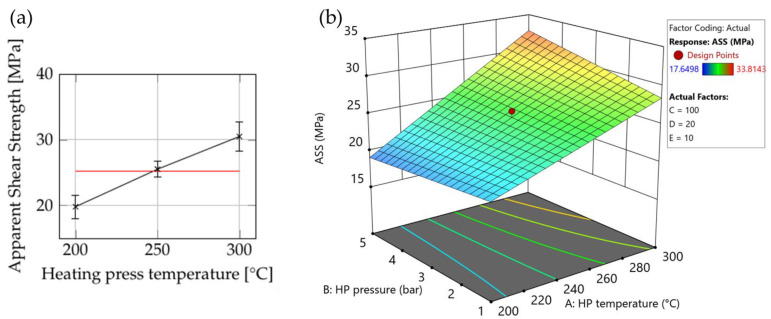
The influence of consolidation settings on the ASS of the plates: (**a**) the influence of heating press temperature; (**b**) surface plot of ASS with the heating press temperature and pressure in the screening trial. Set values for the other parameters: cooling press temperature: 100 °C (factor C); cooling press pressure: 20 bar (factor D); and holding time: 10 s (factor E).

The heating press temperature had a significant impact on the apparent shear strength (see Figure 13 and Table S7). In addition, Figure 13 shows that the combined effect of heating press temperature and pressure—especially at 300 °C—influenced the ASS positively.

This behavior can be explained by examining the degree-of-bonding model [3]. As the temperature increases, the viscosity of the matrix material decreases, promoting higher macromolecular mobility. Consequently, stronger bonding between the layers of the laminate is facilitated as the interlayers diffuse. We assume that, although the heating press temperature was above the glass-transition temperature of the matrix material, a significant amount of time was required to achieve complete bonding, especially at 200 °C heating press temperature.

The highest ASS value measured (33 MPa) fell well below the expected value. The low strength achieved could be attributed to the excessive squeezing of the material, which causes distortion in the inner structure of the composite plate, compromising the strength of the material. Additionally, high void content and/or defects could contribute to the low apparent shear strength. However, further investigation specifically focused on these factors was beyond the scope of this trial.

#### 3.1.5. Screening Trial Discussion

The screening trial provided a first understanding of the key factors that influence the quality parameters of the composite plate, namely heating press temperature and pressure. The cooling press pressure was significant only in relation to density, while cooling press temperature and holding time did not exhibit any significant effect on the quality parameters measured.

Increasing the heating press parameters, specifically the temperature, enhanced the effect of squeeze flow. It promotes better bonding between the layers of the laminate, resulting in improved bonding and compaction, which can potentially lead to a lower void content. However, excessive squeezing is to be avoided, as it results in a significant increase in the projected area, a decrease in thickness of the samples, and an increase in warpage. This could cause problems in later manufacturing stages of the production cell, namely, in the mold of the injection molding machine. Therefore, precise control of the squeeze flow during consolidation is essential. The frame tool shown in Figure 3 partially contributes to achieving this control.

Significant density differences across different areas indicate pressure inhomogeneity during processing of the part. It remains unclear whether the variations in density can be attributed primarily to differences in carbon-fiber content resulting from the squeezing effect or whether void content played a significant role.

The definitive screening test design proved to be effective, providing a general understanding of the influences of process settings on the composite plate. However, due to the presence of aliased two-factor interactions, its usefulness for detailed analysis was found to be limited. Specifically, the design constraints hindered investigation of the combined effects of cooling press temperature and pressure.

In the optimization trial (see Table S2), a split-plot central composite design was implemented. First, the holding time was found to have no significant effect on the quality of the samples and was therefore removed from the design. A fixed cycle time of 180 s was instead used for both presses. Second, the process window of the heating press temperature was redefined to a range between 250 °C and 325 °C. Due to the time-consuming nature of heating and cooling the heating press, this factor was blocked in the experimental design. Although this led to the omission of information concerning the heating press temperature, the significant impact it demonstrated on the quality of the composite plates in the screening trial justified this decision. Third, the range for the heating press pressure was set to between 0.6 bar and 6.5 bar—the minimum and maximum forces achievable by the heating press, respectively. Fourth, the process window for the cooling press temperature was narrowed down to 40–100 °C. Since no significant effect of cooling press temperature was observed in the screening trial, with this adjustment we aimed to investigate whether lowering the temperature range would elicit any additional response in the quality parameters. Finally, the cooling pressure was also redefined to between 10 bar and 85 bar. The selection of 10 bar as the minimum pressure setting was based on its correlation with uneven pressure distribution in the screening trials. The objective was to examine whether this phenomenon would persist in the optimization trials conducted with a frame tool. The 85 bar corresponded to the maximum force the cooling press can exert on the part. These additional limits were chosen to enhance the responsiveness and capture the influence of the cooling press pressure.

### 3.2. Optimization Trials

#### 3.2.1. Thickness

**Figure 14:**
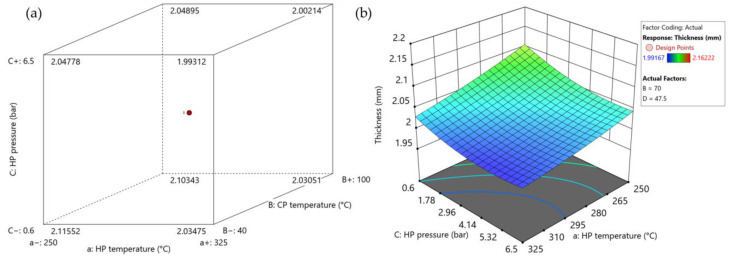
The influences of consolidation settings on part thickness in the optimization trial as summarized in Table S8: (**a**) cube plot representing the influence of heating press (HP) temperature and pressure and cooling press (CP) pressure; (**b**) surface plot showing the interaction between heating press temperature and pressure. Set values for the other parameters: cooling press temperature: 70 °C (factor B); cooling press pressure: 47.5 bar (factor D).

The influence of the consolidation settings on part thickness is plotted in Figure 14a,b, while the ANOVA results are shown in Table S8. Significant influences of heating press temperature, heating press pressure, and cooling press pressure were observed in all areas (see Table 2 and Figure 2) under investigation. In addition, a combined effect of heating press temperature and pressure was detected.

One notable achievement of the frame tool is that it ensured that part thickness remained above 2 mm even at the highest settings. This indicates that production of parts with consistent thickness is possible. Using high heating press temperatures and high heating- and cooling press pressures allows the target thickness of 2 mm to be achieved successfully.

#### 3.2.2. Warpage

**Figure 15:**
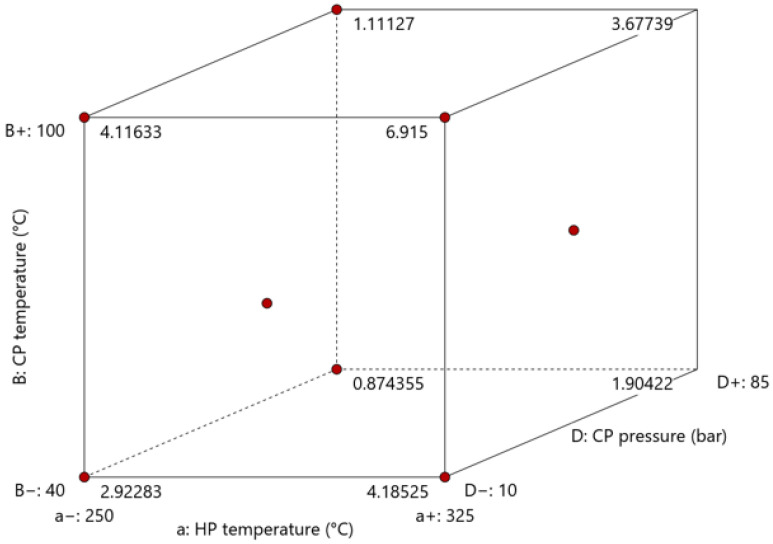
Influence of consolidation settings on warpage in the optimization trials as summarized in Table S9. For the cube plot, the heating press pressure was set to 0.6 bar.

Figure 15 shows the influence of the consolidation settings on warpage, while Table S9 summarizes the ANOVA results. Both indicate that the process settings had no significant influence on warpage.

We conclude that the limitation of squeeze flow led to minimal shape changes in the part, which resulted in a significant reduction in warpage. This is beneficial in terms of process stability, as constant part dimensions are also essential for downstream processing. However, the measurement method employed is unable to assess residual stress. We believe that the part retains a considerable amount of residual stress due to the dynamic nature of the process, but measuring it would require destructive methods.

#### 3.2.3. Void Content

**Figure 16:**
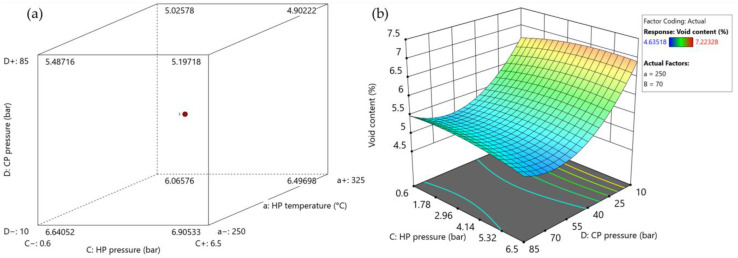
The influence of consolidation settings on the void content in the optimization trial (as summarized in Table S10: (**a**) cube plot representing the influence of heating press temperature and pressure and cooling pressure; (**b**) surface plot showing combined influence of heating pressure and cooling pressure. Set values for the other parameters: heating press temperature: 250 °C (factor a); cooling press temperature: 70°C (factor B).

Figure 16 illustrates the impact of the consolidation settings on the void content, while Table S10 presents the ANOVA results. The effect of heating and cooling press temperature and cooling press pressure is significant. In addition, the combined effects of (i) cooling press temperature and heating pressure and (ii) heating and cooling pressure are significant. The average fiber, matrix, and void volume fraction can be found in Table S11.

Increasing the process settings led to a decrease in void content to some extent. Increasing the cooling press temperature also aids the removal of excessive void content. Employing a slower cooling rate gives the press more time to squeeze out effectively the voids, which results in a reduced void content in the consolidated parts due to lower viscosity and thus in higher molecular mobility. The minimum achieved void content was 4.64%. This value exceeds the upper limit in aerospace applications (1%) [34,35]. However, in some other applications, a maximum of 5% is allowed [36]. The inability to further reduce the void content may be attributed to the presence of inherent voids within the UD tapes themselves, which cannot be eliminated during the manufacturing process.

#### 3.2.4. ASS

**Figure 17:**
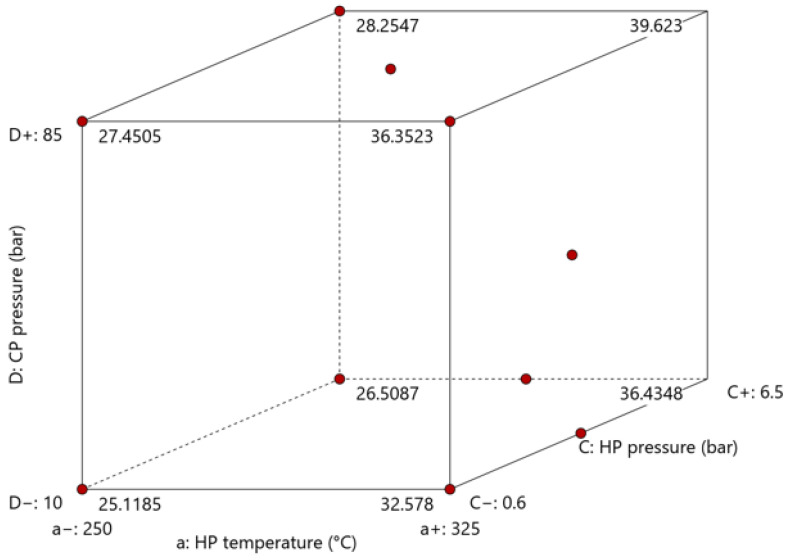
The influence of consolidation settings (as summarized in Table S2) on the apparent shear strength in the optimization trial. For the cube plot, a cooling press temperature of 100 °C was set.

Table S12 summarizes the ANOVA results, while Figure 17 presents a box plot illustrating the influence of process settings on the ASS. The most influential parameter was the heating press temperature, followed by heating pressure and cooling pressure and temperature.

Heating press temperature and pressure also had the greatest influence on the ASS, primarily due to the reduced viscosity of the matrix material. The low strength values obtained at low cooling pressures suggest that the tool does not establish full contact with the part under these conditions. Consequently, pressure and heat are inadequately transferred. This lack of contact is further supported by the void content measurements indicating inadequate quality. Implementation of the frame tool resulted in overall improvements in ASS values. Compared to the maximum bonding achieved in the screening test, an average increase of 23% in ASS was achieved at maximum settings under these conditions, which corresponds to an average value of 41.62 MPa.

#### 3.2.5. Optimization Trials Discussion

In conclusion, the optimization trials resulted in an overall improvement in the quality of the parts produced. The implemented changes, including installation of the frame tool, contributed to maintaining a thickness above 2 mm, minimal changes in the projected area, and a significant enhancement in bonding quality. Controlling the squeeze flow allowed higher process settings to be used, which positively impacted the overall quality of the consolidated plates.

The extended experimental design provided a deeper understanding of the individual process settings and their combined effects. While the influence of the heating press was well understood from the screening test, aliasing of other two-factor interactions meant that understanding of the effects of the cooling press was incomplete.

In most cases, the cooling pressure had a noticeable impact on the quality of the parts, while the cooling press temperature had a minor influence in specific instances. Applying maximum pressure allowed the quality of the consolidated parts to be increased.

Higher void content and lower apparent shear strength values observed at lower settings can be attributed to three main factors. First, the construction of the frame tool consisted of four sections that formed the edges of the consolidated plate. The presence of an 8 mm gap on the right side of the tool allowed the matrix material to flow freely in that direction, resulting in increased thickness, higher void content, and weaker bonding in this area. On the opposite side of the part, we detected a small gap between the components of the frame tool that allowed some material flow, but not to the same extent as on the right side. Second, as hypothesized in the screening test, there may be pressure variations throughout the part. At low cooling pressures, the steel tool plate apparently does not make sufficient contact with the specimen or with the press during consolidation. This is supported by the fact that, at maximum cooling pressure, quality variations between different areas were minimal. Finally, note that the influence of the cooling press on part quality was generally lower than expected. For instance, in the center of the part, the impact of the heating pressure was more significant than that of the cooling pressure, as indicated by the *p*-values in the ANOVA results. This finding was unforeseen, as one would expect a higher impact from increasing the cooling pressure from 10 to 85 bar than from increasing the heating pressure from 0.6 to 6.5 bar.

**Figure 18:**
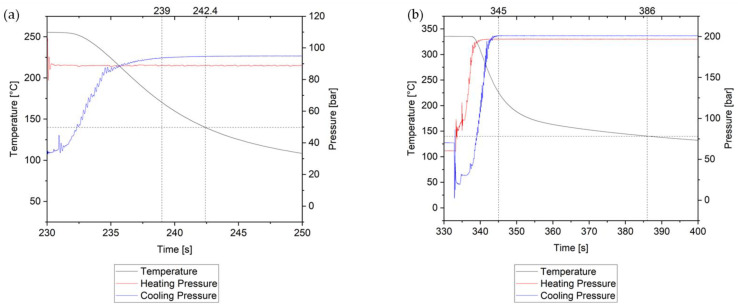
Temperature measurement at the core and the pressure development during consolidation at (**a**) low settings and at (**b**) high settings. The first black vertical line indicates the time at which the cooling press reached its maximum set force, while the second line indicates the time point at which the matrix material reached its T_g_.

To comprehensively assess the influence of the cooling press, tests were conducted to monitor the temperature changes within the core layer of the tape stack (specifically between the 6th and 7th layer) by means of a thermocouple. Additionally, the pressures applied by both the heating and cooling presses were measured and recorded. Figure 18 presents the temperature and pressure profiles during the pressing process, specifically focusing on the stage where the part underwent compression in the cooling press. At the lowest settings (Figure 18a), namely at 250 °C heating press temperature, 0.6 bar heating pressure, 40 °C cooling press temperature and 10 bar cooling pressure, the pressure had built up fully after 239 s of data recording. However, the part reached its glass-transition temperature after 242.4 s in the cooling press. This indicates that during the compression phase in the cooling press, the molten part experienced the defined pressure for only about 3 s. However, at the highest settings (325 °C heating press temperature, 6.5 bar, 100 °C cooling press temperature and 85 bar cooling pressure), the period under full pressure while at a temperature above glass-transition extended to approximately 41 s in the cooling press (Figure 18b). This phenomenon is the primary reason why the cooling press had a limited effect on the void content and ASS results, especially at low heating press temperatures. As indicated by the degree-of-bonding model, adhesion between the layers is a time-dependent process. At the lowest settings, when the part reached its glass-transition temperature in only 3 s, molecular movement between the layers ceased. Our findings demonstrate that this period is too short to achieve adequate bonding between the layers and to eliminate excessive void content.

The numerical optimization tool of the Design Expert software, which combines a desirability function with a hill-climbing technique, can provide suggestions for optimal process settings. Based on our analysis of the results and using this tool, we consider the following process settings to be optimal:

- Heating press temperature: 325 °C;
- Heating pressure: 6.5 bar;
- Cooling press temperature: 100 °C;
- Cooling pressure: 85 bar.

Implementing these settings (the highest in the experimental design) is expected to achieve the desired thickness of 2 mm while minimizing void content and maximizing the apparent shear strength.

## 4. Conclusions

This work focused on optimizing the consolidation of PC/CF tape stacks using a combination of a heating press and a cooling press, with the aim to obtain well-bonded, void-free plates with a target thickness of 2 mm. Two studies were conducted to find optimal process settings and investigate the behavior of the process.

The screening test revealed that increasing the temperature and pressure of the heating press improved part quality, while the cooling press had minimal impact. Excessive squeeze flow resulted in a higher projected area and lower plate thickness than expected.

In the optimization trials, we expanded the experimental design and implemented a frame tool. These modifications had a positive impact on the consolidation process. We thus achieved a plate thickness of 2 mm and a 23% increase in bonding strength compared to the initial trials. The minimum measured void content was 4%. We observed that the cooling press influences the quality of the parts, and that this effect is more pronounced at high settings, where the material remains under pressure and above its T_g_ for a longer period. Based on our findings, we determined that setting the temperature and pressure at both presses as high as possible in this setup is crucial to obtaining parts with optimal properties.

Additional research is required to optimize the process further. We hypothesize that incorporating a fully enclosed frame tool, extending the cycle time, and using a cooling unit that can maintain temperatures above T_g_ of the matrix material would enhance the quality of the parts. Additionally, investigating the process settings’ impact on the degree of crystallinity using a semi-crystalline matrix material would provide valuable insights.

## Figures and Tables

**Table 1:** Properties of the matrix and fiber materials in the UD tape.

Material	Property	Value	Unit
Matrix (Polycarbonate)	Melt mass flow rate	37	g/10 min (300 °C/1.2 kg)
	Density	1190	kg/m^3^
	Glass-transition temperature	145	°C
	Tensile modulus	2400	MPa
	Yield stress	65	MPa, at 50 mm/s
Fiber (Carbon Fiber)	Density	1800	kg/m^3^
	Denier	14,400	den
	Tensile modulus	250	GPa

**Table 2:** Summary of the screening and optimization trials.

Trial	Condition	Experimental Design	Number of Settings	Sample Type	Number of Test Samples Per Setting	Metrics
Screening	No frame tool	Definitive screening design	13	Whole plate	12	Projected area Warpage
				Small samples	14	Thickness Density ASS
Optimization	Frame tool	Split-plot central composite design	26	Whole plate	3	Warpage
				Small samples	9	Thickness Void content ASS

## Data Availability

The data presented in this study are available on request from the corresponding author.

## Supplementary Materials

The following supporting information can be downloaded at: https://www.mdpi.com/article/10.3390/polym15234500/s1, Figure S1: Temperature development at the sample core at set temperatures of (a) 200°C, (b) 250°C and (c) 300°C. The first vertical black line indicates the start of the process, while the second shows the time at which the core has reached the set temperature; Table S1. Experimental design used in the screening test, Table S2. Experimental design used in the optimization test; Table S3. ANOVA for the influence of consolidation settings on the projected area in the screening trial; Table S4. ANOVA for the influence of consolidation settings on the thickness in the screening trial; Table S5. ANOVA for the influence of consolidation settings on the warpage in the screening trial; Table S6. ANOVA for the influence of consolidation settings on the density in the screening trial; Table S7. ANOVA for the influence of consolidation settings on the ASS in the screening trial; Table S8. ANOVA for the influence of consolidation settings on the thickness in the optimization trial; Table S9. ANOVA for the influence of consolidation settings on the warpage in the optimization trial; Table S10. ANOVA for the influence of consolidation settings on the void content in the optimization trial; Table S11. Average fiber, matrix, and void fraction in the optimization trials; Table S12. ANOVA for the influence of consolidation settings on the apparent shear strength in the optimization trial.

## Abbreviations

The following abbreviations are used in this manuscript:

AFP	automated fiber placement
ANOVA	analysis of variance
ASS	apparent shear strength
ATP	automated tape laying
CF/PPS	carbon/polyphenylene sulfide
CP	cooling press
DoE	design of experiments
HP	heating press
PC	polycarbonate
PC/GF	polycarbonate/glass fiber
PC/CF	polycarbonate/carbon fiber
PAEK	polyaryl ether ketone
PEEK	polyetheretherketone
PEKK	polyetherketoneketone
PP	polypropylene
VBO	vacuum-bag-only approach
UD	unidirectional
