# Supplementary material for: Using Heating and Cooling Presses in Combination to Optimize the Consolidation Process of Polycarbonate-Based Unidirectional Thermoplastic Composite Tapes
Source: Polymers (Basel). 2023 Nov 23;15(23):4500. doi: 10.3390/polym15234500 (PMC10707967; doi:10.3390/polym15234500)
Supplement: Supplementary file 1 [file polymers-15-04500-s001.zip › polymers-2650668-supplementary.pdf]

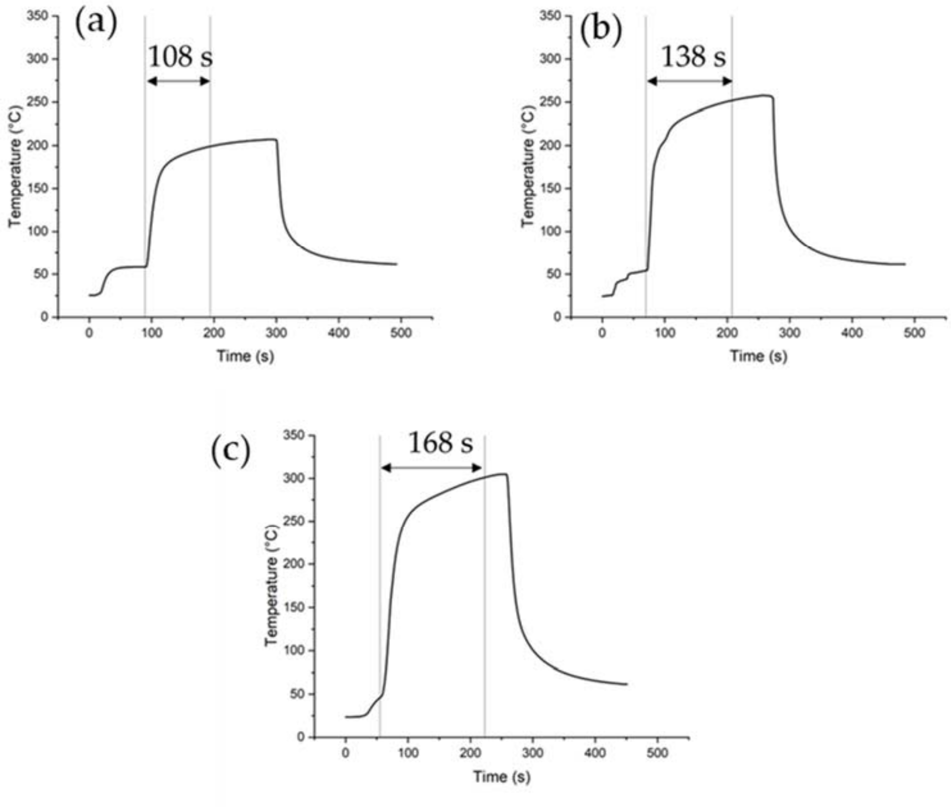

Figure S1. Temperature development at the sample core at set temperatures of a) 200°C, b) 250°C and c) 300°C. The first vertical black line indicates the start of the process, while the second shows the time at which the core has reached the set temperature.

Table S1. Experimental design used in the screening test.

Run	Heating press temperature	Heating press pressure	Cooling press temperature	Cooling press pressure	Holding time
	[°C]	[bar]	[°C]	[bar]	[s]
1	200	5	100	30	5
2	200	1	60	30	10
3	200	5	60	10	15
4	200	3	140	10	5
5	200	1	140	20	15
6	250	1	60	10	5
7	250	5	140	30	15
8	250	3	100	20	10
9	300	5	60	20	5
10	300	5	140	10	10
11	300	3	60	30	15
12	300	1	140	30	5
13	300	1	100	10	15

Table S2. Experimental design used in the optimization test.

Run	Heating press temperature	Cooling press temperature	Heating press temperature	Cooling press pressure
	[°C]	[°C]	bar]	[bar]
1	325	40	3.3	46
2	325	73	6.5	10
3	325	100	0.6	85
4	325	100	6.5	85
5	325	40	0.6	85
6	325	100	3.6	10
7	325	72	0.6	48.3
8	250	100	6.5	10
9	250	40	6.5	85
10	250	40	0.6	10
11	250	100	0.6	85
12	325	40	0.6	10
13	325	100	3.4	47.1
14	325	40	6.5	85
15	288	70	3.6	47.5
16	288	70	3.6	47.5
17	288	70	3.6	47.5
18	250	40	6.5	10
19	250	40	0.6	85
20	250	100	6.5	85
21	250	100	0.6	10
22	288	40	6.5	49.8
23	288	69	0.6	10
24	288	100	3.4	85
25	288	100	6.5	10
26	325	40	6.5	10
27	325	100	6.5	49.4
28	325	68	3.5	85
29	325	100	0.6	10

Table S3. ANOVA for the influence of consolidation settings on the projected area in the screening trial.

Source	Sum of Squares	df	Mean Square	F-value	p-value
**Model**	7.61×10^7	6	1.27×10^7	14.33	0.0025
A-Heat press temperature	5.85×10^7	1	5.85×10^7	66.15	0.0002
B-Heat press pressure	1.03×10^7	1	1.03×10^7	11.65	0.0143
C-Cooling press temperature	1.01×10^5	1	1.01×10^5	0.1138	0.7473
D-Cooling press pressure	44135.27	1	44135.27	0.0499	0.8307
E-Holding time	29403.28	1	29403.28	0.0332	0.8614
AB	7.09×10^6	1	7.09×10^6	8.02	0.0299
**Residual**	5.31×10^6	6	8.85×10^5		
**Cor Total**	8.14×10^7	12			

Table S4. ANOVA for the influence of consolidation settings on the thickness in the screening trial.

Source	Sum of Squares	df	Mean Square	F-value	p-value
**Model**	0.1805	6	0.0301	6.32	0.0205
A-Heat press temperature	0.1476	1	0.1476	31.03	0.0014
B-Heat press pressure	0.0202	1	0.0202	4.25	0.085
C-Cooling press temperature	0.0016	1	0.0016	0.3292	0.587
D-Cooling press pressure	0.001	1	0.001	0.2078	0.6645
E-Holding time	0.0001	1	0.0001	0.0165	0.902
AB	0.01	1	0.01	2.11	0.1968
**Residual**	0.0285	6	0.0048		
**Cor Total**	0.209	12			

Table S5. ANOVA for the influence of consolidation settings on the warpage in the screening trial.

Source	Sum of Squares	df	Mean Square	F-value	p-value
**Model**	1224.52	6	204.09	9.94	0.0066
A-Heating press temperature	941.92	1	941.92	45.88	0.0005

B-Heating press pressure	153.87	1	153.87	7.5	0.0338
C-Cooling press temperature	13.36	1	13.36	0.6506	0.4507
D-Cooling press pressure	18.82	1	18.82	0.9168	0.3753
E-Holding time	0.2758	1	0.2758	0.0134	0.9115
AB	96.28	1	96.28	4.69	0.0735
**Residual**	123.17	6	20.53		
**Cor Total**	1347.69	12			

Table S6. ANOVA for the influence of consolidation settings on the density in the screening trial.

Source	Sum of Squares	df	Mean Square	F-value	p-value
**Model**	0.0024	6	0.0004	17.91	0.0014
A-Heating press temperature	0.0018	1	0.0018	80.44	0.0001
B-Heating press pressure	0.0003	1	0.0003	13.11	0.0111
C-Cooling press temperature	0	1	0	2.09	0.1982
D-Cooling press pressure	0.0002	1	0.0002	8.48	0.0269
E-Holding time	2.49E-07	1	2.49E-07	0.0112	0.9193
AB	0.0001	1	0.0001	3.32	0.1183
**Residual**	0.0001	6	0		

Table S7. ANOVA for the influence of consolidation settings on the ASS in the screening trial.

Source	Sum of Squares	df	Mean Square	F-value	p-value
**Model**	313.03	6	52.17	38.36	0.0002
A-Heating press temperature	285.76	1	285.76	210.1	< 0.0001
B-Heating press pressure	4.45	1	4.45	3.27	0.1206
C-Cooling press temperature	3.4	1	3.4	2.5	0.1651
D-Cooling press pressure	0.0251	1	0.0251	0.0185	0.8964
E-Holding time	1.91	1	1.91	1.41	0.2804
AB	17.49	1	17.49	12.86	0.0116
**Residual**	8.16	6	1.36		
**Cor Total**	321.19	12			

Table S8. ANOVA for the influence of consolidation settings on the thickness in the optimization trial.

Source	Term df	Error df	F-value	p-value
Whole-plot	2	14	81.6	< 0.0001
a-Heating press temperature	1	14	153.11	< 0.0001
a^2	1	14	0.6165	0.4454
Subplot	12	14	22.12	< 0.0001
B-Cooling press temperature	1	14	0.103	0.753
C-Heating press pressure	1	14	97.58	< 0.0001
D-Cooling press pressure	1	14	79.3	< 0.0001
aB	1	14	0.605	0.4496
aC	1	14	6.5	0.0232
aD	1	14	2.45	0.1399
BC	1	14	1.68	0.2155
BD	1	14	1.13	0.306
CD	1	14	1.32	0.2707
B^2	1	14	0.3544	0.5612
C^2	1	14	9.92	0.0071
D^2	1	14	25.46	0.0002

Table S9. ANOVA for the influence of consolidation settings on the warpage in the optimization trial.

Source	Term df	Error df	F-value	p-value
**Whole-plot**	2	14	0.1799	0.8373
a-Heating press temperature	1	14	0.1237	0.7302
a^2	1	14	0.1253	0.7286
**Subplot**	12	14	0.8526	0.6049
B-Cooling press temperature	1	14	0.1901	0.6695
C-Heating press pressure	1	14	0.0022	0.9633
D-Cooling press pressure	1	14	3.94	0.0671
aB	1	14	0.6114	0.4473
aC	1	14	2.45	0.1398
aD	1	14	0.0136	0.9088
BC	1	14	1.17	0.2973
BD	1	14	0.2304	0.6387

CD	1	14	0.5952	0.4533
B^2	1	14	0.0033	0.9551
C^2	1	14	0.1968	0.6641
D^2	1	14	0.1523	0.7023

Table S10. ANOVA for the influence of consolidation settings on the void content in the optimization trial.

Source	Term df	Error df	F-value	p-value
**Whole-plot**	2	14	14.5	0.0004
a-Heating press temperature	1	14	28.41	0.0001
a^2	1	14	0.821	0.3802
**Subplot**	12	14	38.68	< 0.0001
B-Cooling press temperature	1	14	8.22	0.0124
C-Heating press pressure	1	14	0.8414	0.3745
D-Cooling press pressure	1	14	320.56	< 0.0001
aB	1	14	0.4229	0.526
aC	1	14	1.05	0.322
aD	1	14	0.4893	0.4957
BC	1	14	6.01	0.0279
BD	1	14	2.87	0.1123
CD	1	14	11.73	0.0041
B^2	1	14	0.0131	0.9103
C^2	1	14	2.57	0.1312
D^2	1	14	88.79	< 0.0001

Table S11. Average fiber, matrix, and void fraction in the optimization trials.

Run	Fiber volume content [%]	Matrix volume content [%]	Void content [%]
1	51.44	43.51	5.04
2	50.27	43.37	6.36
3	51.44	43.69	4.87
4	51.72	43.65	4.64
5	51.80	43.21	4.99
6	50.14	43.38	6.48
7	50.08	45.02	4.90
8	50.29	43.26	6.46
9	49.86	44.76	5.38

10	49.53	43.65	6.82
11	50.84	43.73	5.43
12	49.93	44.13	5.94
13	50.67	44.38	4.95
14	51.23	43.65	5.13
15	51.21	43.81	4.97
16	51.19	43.75	5.06
17	50.94	44.02	5.05
18	49.94	42.84	7.22
19	50.84	43.72	5.44
20	51.37	43.39	5.24
21	50.26	43.18	6.56
22	50.83	44.16	5.00
23	50.74	42.81	6.45
24	50.93	43.89	5.18
25	50.87	42.68	6.45
26	50.20	42.91	6.89
27	51.30	43.97	4.74
28	50.90	44.11	4.99
29	50.87	43.34	5.79

Table S12. ANOVA for the influence of consolidation settings on the apparent shear strength in the optimization trial.

Source	Term df	Error df	F-value	p-value
**Whole-plot**	2	14	92.87	< 0.0001
a-Heating press temperature	1	14	161.27	< 0.0001
a^2	1	14	0.6403	0.437
**Subplot**	12	14	6.27	0.0009
B-Cooling press temperature	1	14	5.05	0.0413
C-Heating press pressure	1	14	29.35	< 0.0001
D-Cooling press pressure	1	14	16.6	0.0011
aB	1	14	0.2226	0.6444
aC	1	14	3.01	0.1046
aD	1	14	1.03	0.3274
BC	1	14	3.5	0.0826
BD	1	14	0.0007	0.979

CD	1	14	0.1703	0.6861
B^2	1	14	0.049	0.828
C^2	1	14	0.075	0.7882
D^2	1	14	7.26	0.0174
